# Modifiable predictors of atrioventricular block warranting a pacemaker: A cohort study

**DOI:** 10.1371/journal.pone.0356491

**Published:** 2026-09-09

**Authors:** Anna-Sophie Thein, Janet J. Tang, Jean Jacques Noubiap, Thomas A. Dewland, Tor Biering-Sørensen, Gregory M. Marcus

**Affiliations:** 1 Division of Cardiology, Department of Medicine, University of California, San Francisco, California, United States of America; 2 Department of Drug Design and Pharmacology, University of Copenhagen, Copenhagen, Denmark; 3 Center for Translational Cardiology and Pragmatic Randomized Trials, Department of Biomedical Sciences, Faculty of Health and Medical Sciences, University of Copenhagen, Copenhagen, Denmark; 4 Department of Cardiology, Copenhagen University Hospital – Herlev and Gentofte, Copenhagen, Denmark; 5 Steno Diabetes Center Copenhagen, Copenhagen, Denmark; 6 Department of Cardiology, Copenhagen University Hospital – Rigshospitalet, Copenhagen, Denmark; Baylor Scott & White Research Institute, UNITED STATES OF AMERICA

## Abstract

**Background:**

Recent studies focused on atrial fibrillation have demonstrated that the etiology of cardiac arrhythmias may be influenced by modifiable risk factors. It remains unknown if similar predictors are relevant to bradyarrhythmias, the most severe form being atrioventricular (AV) block warranting a pacemaker. This study aimed to identify predictors of AV block warranting a pacemaker.

**Methods:**

We used the California Health Care Access and Information databases to identify Californians ≥18 years who received care in an emergency department, outpatient surgery facility, or hospital in 2005−2020. We examined demographics, comorbidities, lifestyle factors, and air pollution levels as potential predictors. Outcome and covariates were identified using ICD-9, ICD-10, and CPT codes. We used multivariable adjusted Cox proportional models to identify predictors of pacemaker implantation for AV block.

**Results:**

Among 28,640,453 patients, the mean age was 44.2 ± 19.3 years, 54% were female, and 0.3% received a pacemaker for AV block over a mean follow-up of 9.8 ± 4.8 years. In the fully adjusted model, 19 predictors were associated with an increased risk of pacemaker implantation for AV block, including older age, male sex, and cardiovascular comorbidities; modifiable lifestyle factors included heavy alcohol use, use of tobacco, cannabis consumption, and obesity. Asian and Pacific Islanders and Blacks were at lower risk compared to Whites.

**Conclusions:**

This large cohort study identified several modifiable risk factors that may be the focus of future strategies to reduce the risk of AV block warranting a pacemaker.

## Introduction

Pacemakers have been essential in treating bradyarrhythmias for decades, and it is estimated that 1.25 million patients worldwide receive a permanent pacemaker every year [1]. The number of pacemaker implantations and the life span of pacing dependence are increasing [2,3] with the aging population. Atrioventricular (AV) block is a common life-threatening reason for pacemaker implantation [4], but, although pacemakers can provide adequate treatment for the symptoms of AV block, they also have substantial short- and long-term complications, including the risk of thoracic trauma, cardiac injury or tamponade, infection — especially life-threatening endocarditis — and device malfunctions [3,5]. While prevention has been a major focus in other forms of heart disease, such as atrial fibrillation (AF) [6], similar efforts to identify predictors of AV block severe enough to require pacemaker implantation have not yet been pursued. AF and AV block have both been attributed to underlying inflammation and fibrosis of their relevant cardiac tissues (the atria or His-Purkinje system, respectively), suggesting possible overlapping etiologies [7,8]. Renal dysfunction has also been linked to susceptibility to AV conduction abnormalities in prior work [9]. Several common and modifiable risk factors have been associated with AF, including the use of alcohol [10–12], tobacco [13,14], and cannabis [15], physical inactivity [16], and exposure to air pollution [17]. Given the shared underlying pathophysiology, it is reasonable to hypothesize that similar risk factors might enhance the likelihood of developing bradyarrhythmias such as AV block. Identifying potential risk factors for AV block severe enough to warrant a pacemaker may provide essential information to facilitate prevention and identify treatment strategies for patients at risk.

## Methods

### Study design

We conducted a longitudinal cohort study using the HCAI databases that capture every emergency department visit, inpatient hospitalization, and outpatient procedure in California, with the exception of Veterans Affairs and military facilities [18]. Information in these databases includes demographic data and diseases and procedures, with up to 25 diagnoses for each encounter captured using the International Classification of Diseases (ICD)-9th and −10th Revision and Current Procedural Terminology (CPT) codes. Patients are assigned a unique linkage code, which makes it possible to follow them over time and through multiple encounters. Death was ascertained using validated research data sets developed by HCAI that link patient data with the state death statistical master file [19]. Data was accessed from August 2023 to May 2024 and did not include information that could identify individual participants during or after data collection.

### Population

We included adults ≥18 years of age who received care in an emergency department, hospital, or outpatient surgery facility in California from January 1, 2005, to December 31, 2020. We excluded patients if they had no data on age, sex, or race and ethnicity, if their primary residence was outside of California, if they had already received a pacemaker, cardiac resynchronization therapy, or an implantable cardioverter defibrillator at their first visit, or if they had a transcatheter aortic valve replacement or surgical aortic valve replacement up to 30 days prior to pacemaker implantation. Individuals entered the study cohort at their first healthcare encounter and were followed until the incidence of pacemaker implantation, death, or the end of follow-up. They were otherwise censored upon implantation of a cardiac rhythm device that may have been placed for reasons other than bradyarrhythmias, such as devices combined with cardiac resynchronization therapy or an implantable cardioverter defibrillator.

### Outcome

The primary outcome was defined as permanent pacemaker implantation with a concomitant or previous diagnosis of AV block. Pacemaker implantation was identified using ICD-9, ICD-10, and CPT codes, and AV block was identified using ICD-9 and ICD-10 codes (**S1 Table**).

### Predictors

We used data on age, sex, race and ethnicity, and income from the first healthcare encounter. Race and Hispanic ethnicity are reported separately in HCAI, and race was coded as either White or ‘other’ for most individuals with Hispanic ethnicity. Therefore, individuals with Hispanic ethnicity were treated as a distinct group that superseded the coded race. Medical diagnoses and procedures were identified using ICD-9, ICD-10, and CPT codes (**S1 Table**). Covariates were treated as time-updated variables and, once included, carried forward over time. Income was estimated as a state-level quartile derived from the median income by the patient’s five-digit zip code. Air pollution exposure was estimated as the average particulate matter of 2.5 μm or less (PM_2.5_) in 2005 (the beginning of the study period) as measured by the U.S. Environmental Protection Agency monitor station closest to each patient’s zip code [20].

### Statistical analysis

Continuous variables with a normal distribution are presented as means ± SD and were compared using the unpaired 2-sample t-test. Categorical variables are presented as frequencies (percentages) and were compared using the chi-square test. We performed unadjusted and multivariable Cox proportional hazards models to calculate hazard ratios (HR) and their 95% confidence interval (CI) for the incidence of pacemaker implantation with AV block. The proportional hazards assumption was assessed for key covariates using a 5% simple random sample of the data. For PM_2.5_, the assumption was evaluated both graphically, by inspection of cumulative martingale residual plots, and formally, using the supremum test with 1000 simulations; the assumption was not violated (p = 0.876). For sex, the assumption was evaluated graphically using log-log survival plots, which demonstrated parallel curves, consistent with proportional hazards. The covariates included as potential predictors were initially selected based on biological plausibility and previous literature [4,21–28]. Covariates that exhibited 2-sided P < 0.05 in the unadjusted analyses were included in the adjusted model.

If the direction of the relationship between a potential predictor and the outcome changed upon multivariable adjustment, post hoc stepwise forward selection of covariates was performed as an exploratory analysis to identify the variables responsible for the directional change. Stepwise forward selection was not used to select variables for the adjusted model. In addition, as patients could theoretically have a diagnosis of AV block but have received a pacemaker for other reasons, we performed three sensitivity analyses that 1) excluded patients with AF, 2) excluded patients with sick sinus syndrome, and 3) censored patients with AF who underwent catheter ablation of the AV node.

We conducted a Cox proportional hazards model to assess the time-dependent risk of pacemaker implantation with each cumulative number of lifestyle-related exposures (alcohol use, cannabis use, tobacco use, and obesity). We included a time-dependent categorical variable representing each exposure identified during the follow-up period, ranging from 0 (no exposure) to 4 (all four exposures), with 0 as the reference group. These time-dependent covariates change over the course of the study as participants develop new conditions. We also tested this exposure variable as a continuous variable to determine if there was a linear trend across hazard ratios. All other covariates were adjusted for as in prior models.

Data were analyzed using SAS 9.4 (SAS Institute). A 2-sided P < 0.05 was considered to be statistically significant. Dr. Marcus had full access to all the data in the study and takes responsibility for its integrity and the data analysis.

### Data availability

The analytic methods (i.e., program code) are available in the supplemental material. The study authors do not have the authority to share the datasets as they belong to a third party and were obtained as part of a data use agreement between the University of California, San Francisco (UCSF) and the California Department of Health Care Access and Information (HCAI), which precludes sharing data outside UCSF. Access to the dataset can be requested from HCAI by qualified researchers; https://hcai.ca.gov/data/request-data/.

## Results

**Fig 1:**
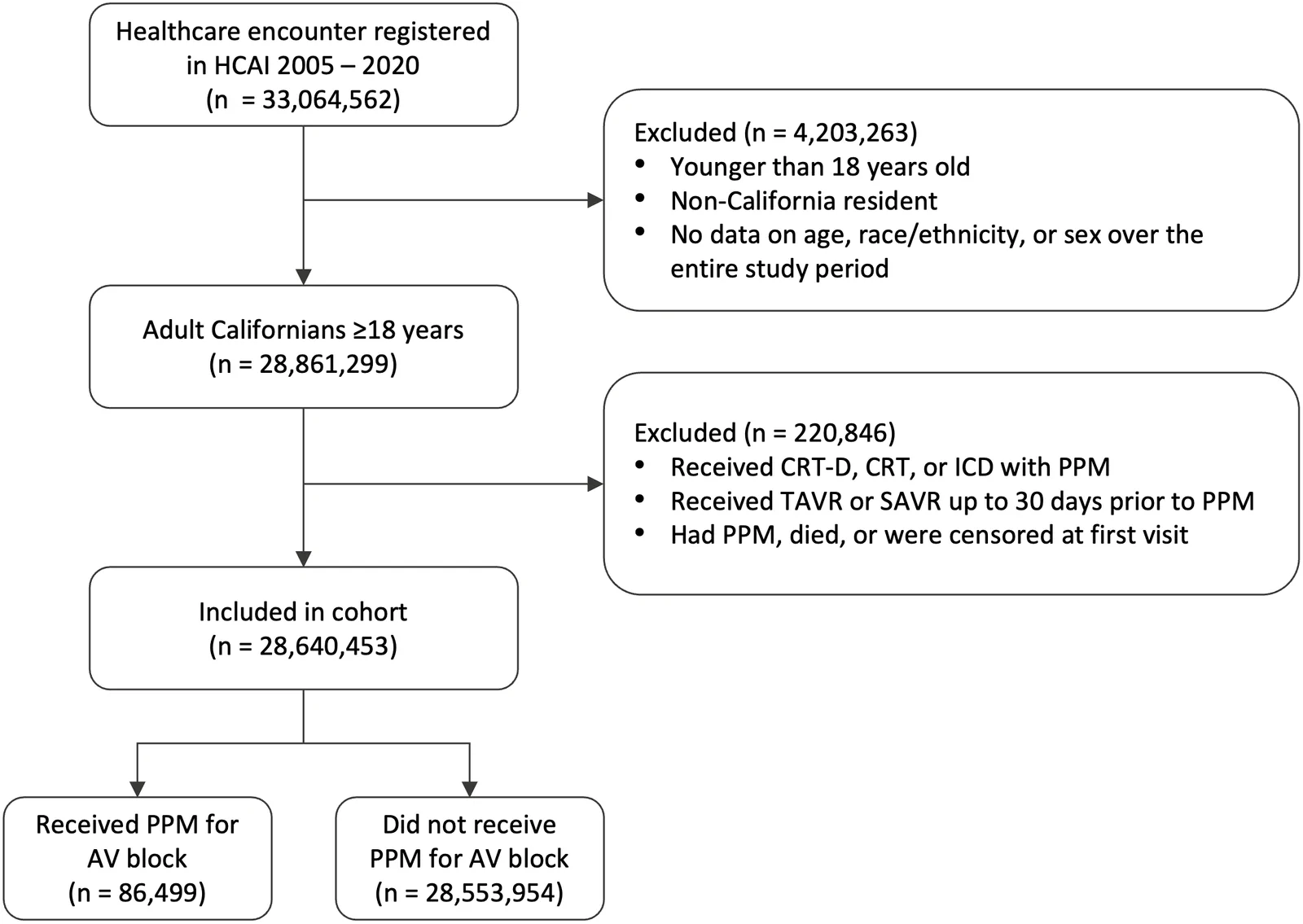
Flow diagram of the study population. Flow diagram showing the stepwise breakdown of the study population to form the final study cohort for analysis after applying inclusion- and exclusion criteria. AV = atrioventricular; CRT = cardiac resynchronization therapy; CRT-D = CRT-defibrillator; ICD = implantable cardioverter-defibrillator; HCAI = Health Care Access and Information; PPM = permanent pacemaker; SAVR = Surgical aortic valve replacement; TAVR = transcatheter aortic valve replacement.

The study included 28,640,453 patients (Fig 1). The mean age was 44.2 ± 19.3 years, 54% were female, and 0.3% received a pacemaker for AV block over a mean follow-up of 9.8 ± 4.8 years. Baseline characteristics are presented in Table 1. Those who received a pacemaker were generally older, more likely to be men, White or American Indian or Alaskan Native individuals, and have cardiovascular comorbidities, of which the most common were hypertension, coronary atherosclerosis, diabetes, and congestive heart failure. Over the study period, 2,808,569 participants died, of whom 241 (0.009%) had received a pacemaker for AV block.

**Table 1:** Baseline characteristics of the study population.

Characteristics	Received a pacemaker for AV block n = 86,499	Did not receive a pacemaker for AV block n = 28,553,954	P value	SMD
Mean age, y	70.6 ± 12.3	44.2 ± 19.3	<.001	1.63
Female	37,789 (43.7)	15,480,013 (54.2)	<.001	−0.21
Race and ethnicity				
White	45,870 (53.0)	12,196,755 (42.7)	<.001	0.21
Black	5315 (6.1)	2,168,949 (7.6)		−0.06
Hispanic	22,565 (26.1)	9,585,707 (33.6)		−0.16
Asian and Pacific Islander	11,621 (13.4)	4,238,225 (14.8)		−0.04
American Indian and Alaskan Native	568 (0.7)	149,950 (0.5)		0.02
Other	560 (0.6)	214,368 (0.8)		−0.01
Income quartile				
1 (lowest)	20,936 (24.2)	7,591,793 (26.6)	<.001	0.07
2	20,280 (23.4)	6,964,050 (24.4)		
3	22,293 (25.8)	7,005,196 (24.5)		
4 (highest)	22,986 (26.6)	6,991,757 (25.5)		
Comorbidities				
Hypertension	77,856 (90.0)	9,867,346 (34.6)	<.001	1.39
Diabetes	37,210 (43.0)	4,336,440 (15.2)	<.001	0.64
Atrial fibrillation	28,779 (33.3)	1,944,234 (6.8)	<.001	0.70
Atrial flutter	9585 (11.1)	376,661 (1.3)	<.001	0.41
Sick sinus syndrome	24,012 (27.8)	242,153 (0.8)	<.001	0.83
Congestive heart failure	34,595 (40.0)	2,131,568 (7.5)	<.001	0.83
Coronary atherosclerosis	41,330 (47.8)	2,585,268 (9.1)	<.001	0.95
Myocardial infarction	21,232 (24.5)	1,546,383 (5.4)	<.001	0.56
Valvular disease, aortic insufficiency	2830 (3.3)	130,675 (0.5)	<.001	0.21
Valvular disease, aortic stenosis	1297 (1.5)	45,895 (0.2)	<.001	0.15
Valvular disease, other	9666 (11.2)	513,066 (1.8)	<.001	0.39
Chronic kidney disease	30,535 (35.3)	2,203,863 (7.7)	<.001	0.71
Lifestyle factors				
Obesity	23,150 (26.8)	4,114,552 (14.4)	<.001	0.31
Lack of physical exercise	34 (0.04)	4189 (0.01)	<.001	0.02
Alcohol use	3456 (4.0)	1,317,991 (4.6)	<.001	−0.03
Tobacco use	11,323 (13.1)	4,346,462 (15.2)	<.001	−0.06
Cannabis use	693 (0.8)	687,411 (2.4)	<.001	−0.13
Air pollutants				
Mean PM_2.5_, μg/m3	13.0 ± 3.8	13.3 ± 3.8	<.001	−0.09

Values are mean ± SD or No. (%). AV = atrioventricular; PM_2.5_ = particulate matter 2.5; SMD = standardized mean difference.

**Fig 2:**
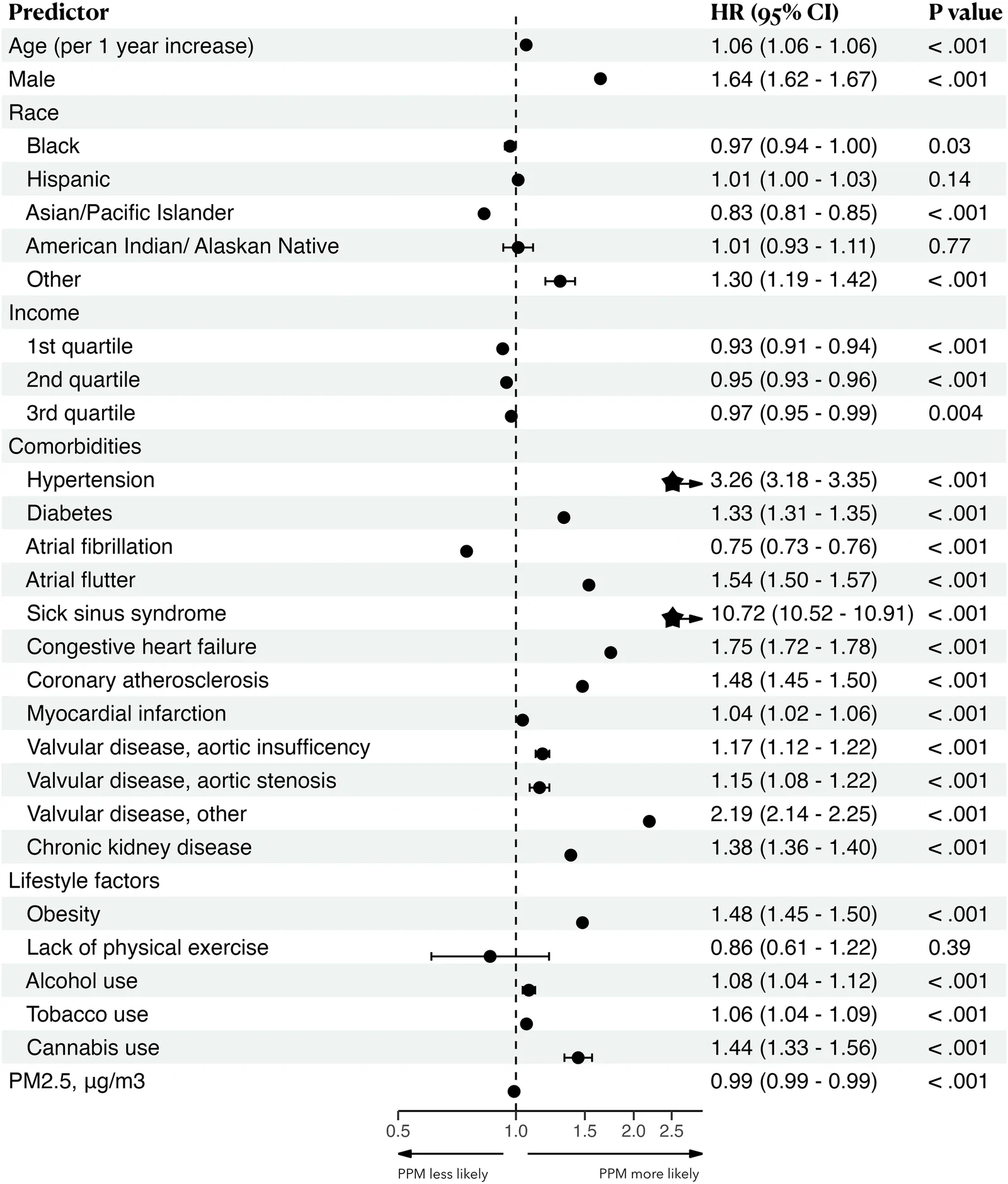
Predictors of permanent pacemaker implantation for atrioventricular block. Forest plot of hazard ratios (HRs) for all predictors included in the multivariable Cox regression model. The model was adjusted for age, sex, race and ethnicity, income, hypertension, diabetes, atrial fibrillation, atrial flutter, sick sinus syndrome, congestive heart failure, coronary atherosclerosis, myocardial infarction, valvular disease, aortic insufficiency, valvular disease, aortic stenosis, valvular disease, other, chronic kidney disease, obesity, lack of physical exercise, alcohol use, tobacco use, cannabis use, and PM_2.5_. Unless otherwise indicated, HRs were interpreted as a hazard for the presence vs absence of each categorical variable or for a 1 unit increase of each continuous variable. Reference group for race and ethnicity was White. Reference group for income was 4^th^ quartile. A star denotes that the HR extended beyond the axis. CI = confidence interval; HR = hazard ratio; PM_2.5_ = particulate matter 2.5; PPM = permanent pacemaker.

Fig 2 shows the hazard ratios for predictors after adjusting for covariates. The predictors associated with the greatest increase in risk were, in order of magnitude, sick sinus syndrome, hypertension, congestive heart failure, and male sex. Statistically significant modifiable risk factors associated with an increased risk included alcohol use disorders, use of tobacco and cannabis, and obesity.

Before multivariable adjustment, the risk of pacemaker implantation was higher in patients with AF (HR 11.64, 95% CI 11.48–11.81, p < .001). However, this relationship was reversed after adjustment (HR 0.75, 95% CI 0.734–0.762, p < .001). Stepwise forward selection showed that adjusting for sick sinus syndrome, age, and hypertension were primarily responsible for the change from higher to lower risk of pacemaker implantation (**S2 Table**).

The time-dependent Cox model assessing the effect of a cumulative number of lifestyle-related exposures (alcohol use, cannabis use, tobacco use, and obesity) showed that the risk of pacemaker implantation increased by 25% with each additional exposure (HR 1.25, 95% CI 1.240–1.269, p < .001).

Upon excluding patients with AF or sick sinus syndrome, or censoring patients with AF who received intracardiac catheter ablation of the AV node, the results did not reveal any meaningful changes in the magnitudes or directions of the observed relationships (**S3 Table**).

## Discussion

In a large cohort of patients who received care in an emergency department, hospital, or outpatient surgery facility in California, we found that older age, male sex, having a higher income, and having more cardiovascular comorbidities increased the risk of undergoing pacemaker implantation for AV block, whereas Asian and Pacific Islander and Black patients were at lower risk than White patients. Several lifestyle factors significantly increased this risk even after adjusting for these other covariates, including heavy alcohol use, use of tobacco, cannabis consumption, and obesity.

Our findings are consistent with previous studies, which similarly have reported these risk factors of age, demographics, and established cardiovascular comorbidities as risk factors of cardiac conduction disease [16] or AV block [4,29,30], which may provide some validation of our methods. Whereas previous studies included thousands of individuals, of which hundreds had the primary outcome, this current study included millions of patients, of which tens of thousands had the outcome. As this is, to our knowledge, the largest study on the subject, we were able to detect independent and positive associations with alcohol, tobacco, and cannabis use and obesity, each representing novel predictors of pacemaker implantations for AV block not previously reported.

Studies have linked alcohol, tobacco, and cannabis use, as well as obesity, to increased oxidative stress, inflammation, and fibrosis — factors that also play a role in the etiology of AV block [31–35]. Additionally, pre-clinical studies have found potential mechanisms of alcohol-induced heart damage [36], such as interference with excitation-contraction coupling mechanisms [37,38] and impairment of myocyte regeneration [39] – potential mechanisms that might influence conduction disorders.

However, previous literature on the association between the use of these substances with the risk of AV block is sparse and mainly limited to case reports of acute use [40–44]. In that setting, AV block is more likely to represent aberrations in vagal tone rather than a chronic structural disease of the conduction system. Whereas the former is reversible, it is only the latter type of AV block that warrants a pacemaker. Two previous cohort studies found no association between chronic alcohol consumption and the risk of bradyarrhythmia [4,45], however, these studies ascertained alcohol use from a questionnaire of self-reported alcohol consumption, which may be subject to both recall and social desirability bias [46–48]. In contrast, the HCAI databases used in the current study rely on healthcare professional coding (rather than self-report), and, although susceptible to incomplete documentation, we would expect this to manifest as underreporting of what should otherwise be specific ascertainment of alcohol, tobacco, and cannabis use, resulting in a loss of power to detect actual relationships rather than cause false positive results. This may suggest that the association between alcohol, tobacco, and cannabis use and the risk of pacemaker implantation could be even more marked than what was measured in our study. Lastly, as prior studies of chronic alcohol and tobacco use [4,28,45] were conducted in relatively smaller cohorts, insufficient power may explain the prior negative findings. The results from the time-dependent Cox model of cumulative lifestyle-related exposures highlight the additive effect of multiple lifestyle-related risk factors. This both emphasizes the importance of targeting every factor possible, but it also points to the potential decrease in risk that modifying just one risk factor could have on the risk of pacemaker implantation. Given the potential for impactful interventions on both an individual and policy level, these novel findings of positive associations with substance use, if confirmed in future studies, might be used to inform future clinical practice guidelines. To our knowledge, no prior study has examined chronic cannabis use as a risk factor for conduction disease or AV block. Despite recreational cannabis now being legal in 23 states, where more than 100 million Americans reside, rigorous studies on the actual health effects are sparse and this new finding highlights an important aspect for future studies.

We found a very small but negative relation between PM_2.5_ and pacemaker implantation, which might appear to contradict the growing epidemiological evidence that links air pollution to an increased risk of cardiac arrhythmias [49]. Of note, those prior studies have largely focused on tachyarrhythmias, whereas this study sought to identify predictors of the most severe form of bradyarrhythmia. As particulate matter exposure in the current study was based on a zip code level average in 2005, we acknowledge that our assessment of air pollution does not fully reflect individual and time-dependent exposure, which may yet reveal important associations in future investigations.

AF was associated with an unexpected lower risk of incident pacemaker placement in patients with AV block. Before multivariable adjustment, AF was associated with an increased risk of pacemaker implantation, and this apparently protective relationship did not manifest until after multivariable adjustment. One possible explanation is the diagnostic electrocardiographic characteristics of AV block requiring a pacemaker, which may be less obvious in the setting of AF. For example, infranodal AV block could manifest as slower AF. It is also possible that AF itself is able to help maintain a sufficiently high heart rate in the setting of severe AV conduction disease that pacemakers are less often needed.

### Limitations

We acknowledge that our study has several limitations. As with all studies using diagnostic and procedural coding for ascertainment, our study relied on accurate provider documentation. However, prior validation studies using similar databases have shown less than 2% false positives and negatives for pacemaker implantations identified using diagnostic coding, and a high validity for cardiac diagnoses [50–55]. As with all studies relying on ICD-10 codes, there is a risk of misclassification bias, including potential differential classification by race or ethnicity. However, as this study did not analyze lifestyle-related outcomes stratified by race or ethnicity (and the findings remained consistent after adjusting for race and ethnicity), such misclassification is unlikely to meaningfully affect the interpretation of the results. Indeed, while a focus on individualized risk assessment was not pursued in the current study, future research investigating interactions by various patient characteristics, such as those of different sex, age, race/ethnicity, or income, will be important to inform clinically relevant risk counselling and stratification. Given a reliance on ICD-9, ICD-10, and CPT codes, we were unable to comment on the dose-response relationships between each predictor and the risk of pacemaker implantation. Thus, our findings related to lifestyle-related risk factors lack the granularity to determine the exact severity, frequency, or duration of substance use and obesity. Because of the administrative nature of the data, our primary outcome may be influenced by access to health care, which might explain the apparently “protective” effect of lower income on pacemaker implantation. Of note, the other associations were observed despite adjusting for income as a covariate. Although the HCAI databases do not include ambulatory clinic data, they should have captured every pacemaker implantation in California over the 15-year study period. Similarly, all deaths should be available in the Social Security Death Index, and analyses accounted for the competing risk of death. Given the exploratory nature of these analyses, our examination of many potential predictors resulted in multiple comparisons, which may lead to statistically significant associations arising due to chance. However, in such a large dataset, the directions of the relationships are arguably the most important, and observed associations fit with the biological plausibility and previous literature. While we cannot exclude reverse causality (for example, bradycardia leading to reduced physical fitness prior to the need for a pacemaker), it would appear unlikely that such phenomena would explain relationships with tobacco smoking or cannabis. Finally, as this was an observational study, we cannot exclude residual or unmeasured confounding, making confident causal inferences inappropriate.

## Conclusion

In this large longitudinal cohort study, we found that certain demographic characteristics, multiple established cardiovascular risk factors, and several common modifiable lifestyle factors predicted AV block warranting a pacemaker. These data suggest that minimizing alcohol, avoiding tobacco and cannabis use, and weight loss among the obese are potential strategies to reduce the risk of AV block warranting a pacemaker.

## Supporting information

S1 Table International Classification of Diseases Ninth (ICD-9) and Tenth (ICD-10) Revision Diagnosis and Procedure codes and Current Procedural Terminology (CPT) codes used for disease and procedure identification. (DOCX)

S2 Table Stepwise forward selection of covariates responsible for change from higher to lower risk of pacemaker implantation after multivariable adjustment in patients with atrial fibrillation. (DOCX)

S3 Table Sensitivity analyses comparing the hazard ratios of pacemaker implantation when including all patients vs excluding patients with sick sinus syndrome vs excluding patients with atrial fibrillation. (DOCX)

## References

[1] Carrión-Camacho MR, Marín-León I, Molina-Doñoro JM, González-López JR. Safety of permanent pacemaker implantation: a prospective study. J Clin Med. 2019;8(1):35. doi: 10.3390/jcm8010035 30609668 PMC6352172

[2] Raatikainen MJP, Arnar DO, Merkely B, Nielsen JC, Hindricks G, Heidbuchel H, et al. A decade of information on the use of cardiac implantable electronic devices and interventional electrophysiological procedures in the European Society of Cardiology Countries: 2017 report from the European Heart Rhythm Association. Europace. 2017;19:ii1–90. doi: 10.1093/europace/eux258 28903470

[3] Polyzos KA, Konstantelias AA, Falagas ME. Risk factors for cardiac implantable electronic device infection: a systematic review and meta-analysis. Europace. 2015;17(5):767–77. doi: 10.1093/europace/euv053 25926473

[4] Kerola T, Eranti A, Aro AL, Haukilahti MA, Holkeri A, Junttila MJ, et al. Risk factors associated with atrioventricular block. JAMA Netw Open. 2019;2(5):e194176. doi: 10.1001/jamanetworkopen.2019.4176 31125096 PMC6632153

[5] Udo EO, Zuithoff NPA, van Hemel NM, de Cock CC, Hendriks T, Doevendans PA, et al. Incidence and predictors of short- and long-term complications in pacemaker therapy: the FOLLOWPACE study. Heart Rhythm. 2012;9(5):728–35. doi: 10.1016/j.hrthm.2011.12.014 22182495

[6] Chung MK, Eckhardt LL, Chen LY, Ahmed HM, Gopinathannair R, Joglar JA, et al. Lifestyle and risk factor modification for reduction of atrial fibrillation: a scientific statement from the American Heart Association. Circulation. 2020;141(16):e750–72. doi: 10.1161/CIR.0000000000000748 32148086

[7] Harada M, Nattel S. Implications of inflammation and fibrosis in atrial fibrillation pathophysiology. Card Electrophysiol Clin. 2021;13(1):25–35. doi: 10.1016/j.ccep.2020.11.002 33516403

[8] Zoob M, Smith KS. The aetiology of complete heart-block. Br Med J. 1963;2(5366):1149–53. doi: 10.1136/bmj.2.5366.1149 14060910 PMC1874084

[9] Parsova KE, Hayiroglu MI, Pay L, Cinier G, Gurkan K. Long-term follow-up of patients with drug-related atrioventricular block without a need of permanent pacemaker during index hospitalization. Egypt Heart J. 2022;74(1):56. doi: 10.1186/s43044-022-00297-3 35913636 PMC9343480

[10] Dukes JW, Dewland TA, Vittinghoff E, Olgin JE, Pletcher MJ, Hahn JA, et al. Access to alcohol and heart disease among patients in hospital: observational cohort study using differences in alcohol sales laws. BMJ. 2016;353:i2714. doi: 10.1136/bmj.i2714 27301557 PMC4908314

[11] Whitman IR, Agarwal V, Nah G, Dukes JW, Vittinghoff E, Dewland TA. Alcohol abuse and cardiac disease. J Am Coll Cardiol. 2017;69:13–24. doi: 10.1016/j.jacc.2016.10.048 28057245 PMC5226115

[12] Marcus GM, Vittinghoff E, Whitman IR, Joyce S, Yang V, Nah G. Acute consumption of alcohol and discrete atrial fibrillation events. Ann Intern Med. 2021;174:1503–9. doi: 10.7326/M21-0228 34461028

[13] Chamberlain AM, Agarwal SK, Folsom AR, Duval S, Soliman EZ, Ambrose M, et al. Smoking and incidence of atrial fibrillation: results from the Atherosclerosis Risk in Communities (ARIC) study. Heart Rhythm. 2011;8(8):1160–6. doi: 10.1016/j.hrthm.2011.03.038 21419237 PMC3139831

[14] Groh CA, Vittinghoff E, Benjamin EJ, Dupuis J, Marcus GM. Childhood tobacco smoke exposure and risk of atrial fibrillation in adulthood. J Am Coll Cardiol. 2019;74(13):1658–64. doi: 10.1016/j.jacc.2019.07.060 31558248 PMC6768078

[15] Lin AL, Nah G, Tang JJ, Vittinghoff E, Dewland TA, Marcus GM. Cannabis, cocaine, methamphetamine, and opiates increase the risk of incident atrial fibrillation. Eur Heart J. 2022;43(47):4933–42. doi: 10.1093/eurheartj/ehac558 36257330

[16] Frimodt-Møller EK, Soliman EZ, Kizer JR, Vittinghoff E, Psaty BM, Biering-Sørensen T, et al. Lifestyle habits associated with cardiac conduction disease. Eur Heart J. 2023;44(12):1058–66. doi: 10.1093/eurheartj/ehac799 36660815 PMC10226753

[17] Shahrbaf MA, Akbarzadeh MA, Tabary M, Khaheshi I. Air pollution and cardiac arrhythmias: a comprehensive review. Curr Probl Cardiol. 2021;46(3):100649. doi: 10.1016/j.cpcardiol.2020.100649 32839041

[18] hcai.ca.gov. Fact Sheet: How is OSHPD data used? Available from: https://hcai.ca.gov/wp-content/uploads/2020/10/AboutOSHPD_Data.pdf

[19] Research Data Request Information. In: HCAI [Internet]. [cited 20 Apr 2024]. Available from: https://hcai.ca.gov/data/request-data/research-data-request-information/

[20] Trinh P, Allerup JAC, Li S, Ko J, Chen J, Linos E, et al. Association between yearlong air pollution and moderate-severe atopic dermatitis: a United States cross-sectional claims analysis. JAAD Int. 2023;13:4–6. doi: 10.1016/j.jdin.2023.04.017 37592975 PMC10430148

[21] Kojic EM, Hardarson T, Sigfusson N, Sigvaldason H. The prevalence and prognosis of third-degree atrioventricular conduction block: the Reykjavik study. J Intern Med. 1999;246(1):81–6. doi: 10.1046/j.1365-2796.1999.00521.x 10447229

[22] Rudbeck-Resdal J, Christiansen MK, Johansen JB, Nielsen JC, Bundgaard H, Jensen HK. Aetiologies and temporal trends of atrioventricular block in young patients: a 20-year nationwide study. Europace. 2019;21(11):1710–6. doi: 10.1093/europace/euz206 31424500 PMC6826204

[23] Cheng S, Keyes MJ, Larson MG, McCabe EL, Newton-Cheh C, Levy D, et al. Long-term outcomes in individuals with prolonged PR interval or first-degree atrioventricular block. JAMA. 2009;301(24):2571–7. doi: 10.1001/jama.2009.888 19549974 PMC2765917

[24] Movahed M-R, Hashemzadeh M, Jamal MM. Increased prevalence of third-degree atrioventricular block in patients with type II diabetes mellitus. Chest. 2005;128(4):2611–4. doi: 10.1378/chest.128.4.2611 16236932

[25] Tadros R, Ton A-T, Fiset C, Nattel S. Sex differences in cardiac electrophysiology and clinical arrhythmias: epidemiology, therapeutics, and mechanisms. Can J Cardiol. 2014;30(7):783–92. doi: 10.1016/j.cjca.2014.03.032 24970790

[26] Dewland TA, Soliman EZ, Davis BR, Magnani JW, Yamal J-M, Piller LB, et al. Effect of the Antihypertensive and Lipid-Lowering Treatment to Prevent Heart Attack Trial (ALLHAT) on Conduction System Disease. JAMA Intern Med. 2016;176(8):1085–92. doi: 10.1001/jamainternmed.2016.2502 27367818

[27] Frimodt-Møller EK, Vittinghoff E, Kaur G, Biering-Sørensen T, Soliman EZ, Marcus GM. Association between intensive vs standard blood pressure control and incident left ventricular conduction disease: a post hoc analysis of the SPRINT Randomized Clinical Trial. JAMA Cardiol. 2023;8(6):612–6. doi: 10.1001/jamacardio.2023.0845 37133829 PMC10157506

[28] Frimodt-Møller EK, Gottdiener JS, Soliman EZ, Kizer JR, Vittinghoff E, Psaty BM, et al. Inflammation and incident conduction disease. J Am Heart Assoc. 2023;12(1):e027247. doi: 10.1161/JAHA.122.027247 36565176 PMC9973568

[29] Shan R, Ning Y, Ma Y, Liu S, Wu J, Fan X, et al. Prevalence and risk factors of atrioventricular block among 15 million Chinese health examination participants in 2018: a nation-wide cross-sectional study. BMC Cardiovasc Disord. 2021;21(1):289. doi: 10.1186/s12872-021-02105-3 34116630 PMC8194203

[30] Kataoka S, Kobayashi Y, Isogai T, Tanno K, Fukamizu S, Watanabe N, et al. Permanent pacemaker implantation and its predictors in patients admitted for complete atrioventricular block: a report from the Tokyo Cardiovascular Care Unit Network multi-center registry. Heart Vessels. 2020;35(11):1573–82. doi: 10.1007/s00380-020-01642-9 32500173

[31] Goette A, Lendeckel U, Kuchenbecker A, Bukowska A, Peters B, Klein HU, et al. Cigarette smoking induces atrial fibrosis in humans via nicotine. Heart. 2007;93(9):1056–63. doi: 10.1136/hrt.2005.087171 17395670 PMC1955003

[32] Yanbaeva DG, Dentener MA, Creutzberg EC, Wesseling G, Wouters EFM. Systemic effects of smoking. CHEST. 2007;131:1557–66. doi: 10.1378/chest.06-2179 17494805

[33] Pabon MA, Manocha K, Cheung JW, Lo JC. Linking arrhythmias and adipocytes: insights, mechanisms, and future directions. Front Physiol. 2018;9:1752. doi: 10.3389/fphys.2018.01752 30568603 PMC6290087

[34] Rajesh M, Mukhopadhyay P, Bátkai S, Patel V, Saito K, Matsumoto S, et al. Cannabidiol attenuates cardiac dysfunction, oxidative stress, fibrosis, and inflammatory and cell death signaling pathways in diabetic cardiomyopathy. J Am Coll Cardiol. 2010;56(25):2115–25. doi: 10.1016/j.jacc.2010.07.033 21144973 PMC3026637

[35] Voskoboinik A, Prabhu S, Ling L-H, Kalman JM, Kistler PM. Alcohol and atrial fibrillation: a sobering review. J Am Coll Cardiol. 2016;68(23):2567–76. doi: 10.1016/j.jacc.2016.08.074 27931615

[36] Fernández-Solà J, Planavila Porta A. New treatment strategies for alcohol-induced heart damage. Int J Mol Sci. 2016;17(10):1651. doi: 10.3390/ijms17101651 27690014 PMC5085684

[37] Thomas AP, Sass EJ, Tun-Kirchmann TT, Rubin E. Ethanol inhibits electrically-induced calcium transients in isolated rat cardiac myocytes. J Mol Cell Cardiol. 1989;21(6):555–65. doi: 10.1016/0022-2828(89)90821-3 2778807

[38] Nicolás JM, Rubin E, Thomas AP. Ethanol and cocaine cause additive inhibitory effects on the calcium transients and contraction in single cardiomyocytes. Alcohol Clin Exp Res. 1996;20(6):1077–82. doi: 10.1111/j.1530-0277.1996.tb01949.x 8892530

[39] Fernández-Solà J. Cardiovascular risks and benefits of moderate and heavy alcohol consumption. Nat Rev Cardiol. 2015;12(10):576–87. doi: 10.1038/nrcardio.2015.91 26099843

[40] Richards JR, Blohm E, Toles KA, Jarman AF, Ely DF, Elder JW. The association of cannabis use and cardiac dysrhythmias: a systematic review. Clin Toxicol (Phila). 2020;58(9):861–9. doi: 10.1080/15563650.2020.1743847 32267189

[41] Yahud E, Paul G, Rahkovich M, Vasilenko L, Kogan Y, Lev E, et al. Cannabis induced cardiac arrhythmias: a case series. Eur Heart J Case Rep. 2020;4(6):1–9. doi: 10.1093/ehjcr/ytaa376 33442601 PMC7793045

[42] Van Keer JM. Cannabis-induced third-degree AV block. Case Rep Emerg Med. 2019;2019:5037356. doi: 10.1155/2019/5037356 31637064 PMC6766110

[43] Thienel M, Woernle M, Sinner MF, Brunner S, Petzold T. Atrioventricular block grade III in the context of acute alcohol intake. Eur J Emerg Med. 2021;28(1):75–6. doi: 10.1097/MEJ.0000000000000734 32976311

[44] van Stigt AH, Overduin RJ, Staats LC, Loen V, van der Heyden MAG. A heart too drunk to drive; AV block following acute alcohol intoxication. Chin J Physiol. 2016;59(1):1–8. doi: 10.4077/CJP.2016.BAE364 26875557

[45] Tu SJ, Gallagher C, Elliott AD, Linz D, Pitman BM, Hendriks JML, et al. Alcohol intake and bradyarrhythmia risk: a cohort study of 407 948 individuals. Europace. 2022;24(9):1469–74. doi: 10.1093/europace/euac007 35178566 PMC9559907

[46] Livingston M, Callinan S. Underreporting in alcohol surveys: whose drinking is underestimated? J Stud Alcohol Drugs. 2015;76(1):158–64. doi: 10.15288/jsad.2015.76.158 25486405

[47] Midanik L. The validity of self‐reported alcohol consumption and alcohol problems: A literature review. Br J Addict. 1982. [cited 28 Mar 2024]. Available from: https://onlinelibrary-wiley-com.ucsf.idm.oclc.org/doi/10.1111/j.1360-0443.1982.tb02469.x 10.1111/j.1360-0443.1982.tb02469.x 6762224

[48] Davis CG, Thake J, Vilhena N. Social desirability biases in self-reported alcohol consumption and harms. Addict Behav. 2010;35(4):302–11. doi: 10.1016/j.addbeh.2009.11.001 19932936

[49] Zhang S, Lu W, Wei Z, Zhang H. Air pollution and cardiac arrhythmias: from epidemiological and clinical evidences to cellular electrophysiological mechanisms. Front Cardiovasc Med. 2021;8:736151. doi: 10.3389/fcvm.2021.736151 34778399 PMC8581215

[50] Bradshaw PJ, Stobie P, Knuiman MW, Briffa TG, Hobbs MST. Trends in the incidence and prevalence of cardiac pacemaker insertions in an ageing population. Open Heart. 2014;1(1):e000177. doi: 10.1136/openhrt-2014-000177 25512875 PMC4265147

[51] Teng T-HK, Finn J, Hung J, Geelhoed E, Hobbs M. A validation study: how effective is the Hospital Morbidity Data as a surveillance tool for heart failure in Western Australia? Aust N Z J Public Health. 2008;32(5):405–7. doi: 10.1111/j.1753-6405.2008.00269.x 18959540

[52] McCormick N, Lacaille D, Bhole V, Avina-Zubieta JA. Validity of myocardial infarction diagnoses in administrative databases: a systematic review. PLoS One. 2014;9(3):e92286. doi: 10.1371/journal.pone.0092286 24682186 PMC3969323

[53] Saczynski JS, Andrade SE, Harrold LR, Tjia J, Cutrona SL, Dodd KS, et al. A systematic review of validated methods for identifying heart failure using administrative data. Pharmacoepidemiol Drug Saf. 2012;21 Suppl 1(0 1):129–40. doi: 10.1002/pds.2313 22262599 PMC3808171

[54] Roumie CL, Mitchel E, Gideon PS, Varas-Lorenzo C, Castellsague J, Griffin MR. Validation of ICD-9 codes with a high positive predictive value for incident strokes resulting in hospitalization using Medicaid health data. Pharmacoepidemiol Drug Saf. 2008;17(1):20–6. doi: 10.1002/pds.1518 17979142

[55] Davis LA, Mann A, Cannon GW, Mikuls TR, Reimold AM, Caplan L. Validation of diagnostic and procedural codes for identification of acute cardiovascular events in US veterans with rheumatoid arthritis. EGEMS (Wash DC). 2014;1(3):1023. doi: 10.13063/2327-9214.1023 25848582 PMC4371488

