# Supplementary material for: Modifiable predictors of atrioventricular block warranting a pacemaker: A cohort study
Source: PLoS One. 2026 Sep 9;21(9):e0356491. doi: 10.1371/journal.pone.0356491 (PMC13557407; doi:10.1371/journal.pone.0356491)
Supplement: Supplementary file 1 [file pone.0356491.s001.docx]

**S1 Table.** International Classification of Diseases Ninth (ICD-9) and Tenth (ICD-10) Revision Diagnosis and Procedure codes and Current Procedural Terminology (CPT) codes Used for Disease and Procedure Identification

| **Diagnosis*** | **ICD-9** | **ICD-10** | **CPT** |
| --- | --- | --- | --- |
| **Outcomes** | | | |
| Atrioventricular block | 426.0, 426.10, 426.12, 426.13 | I44.1, I44.2, I44.3, I44.30, I44.39 |  |
| Permanent pacemaker implantation | 37.80, 37.81, 37.82, 37.83 | Z95.0, 02H60JZ, 02H63JZ, 02H64JZ, 02HK0JZ, 02HK3JZ, 02HK3NZ, 02HK4JZ, 02HL0JZ, 02HL3JZ, 02HL4JZ, 0JH604Z, 0JH605Z, 0JH606Z, 0JH634Z, 0JH635Z, 0JH636Z, 0JH804Z, 0JH805Z, 0JH806Z, 0JH834Z, 0JH835Z, 0JH836Z | 33206, 33207, 33208 |
| **Predictors** | | | |
| Alcohol related disorders | 305.0X | F10X |  |
| Atrial fibrillation | 427.31 | I48.1X, I48.2X, I48.91 |  |
| Atrial flutter | 427.32 | I48.3, I48.4, I48.92 |  |
| Cannabis abuse | 304.3X | F12X |  |
| Cardiac resynchronization defibrillator (CRT-D) | 00.51 | 0JH609Z, 0JH639Z, 0JH809Z, 0JH839Z |  |
| Cardiac resynchronization therapy (CRT) | 00.50, 00.52 | 02H43JZ, 02H44JZ, 02HL0JZ, 02HL3JZ, 0JH607Z, 0JH637Z, 0JH807Z, 0JH837Z | 33225 |
| Catheter ablation |  |  | 93650 |
| Chronic kidney disease | 585.X | I12.X, I13.X, N18.X |  |
| Congestive heart failure | 428X | I50.X |  |
| Coronary atherosclerosis | 414.0X | I25X |  |
| Diabetes | 250.X | E10.X, E11.X, E13.X, E14.X |  |
| Epicardial electrode |  |  | 33202, 33203, 33236 |
| Hypertension | 401.X, 402.X, 403.X, 404.X, 405X | I10.X, I11.X, I12.X, I13.X, I15X |  |
| Implantable cardioverter-defibrillator (ICD) |  | Z95.810,  02H43KZ, 02H63KZ, 02HK3KZ, 02HN0KZ, 0JH608Z, 0JH638Z, 0JH808Z, 0JH838Z | 33216, 33217, 33230, 33231, 33240, 33249, 33249, 33271 |
| Lack of physical exercise | Z72.3 | V690 |  |
| Myocardial infarction | 410X, 412X | I21X, I22X, I25.2, I25.6 |  |
| Obesity | 278.00, 278.01, 278.02 | E66X |  |
| Revisions, repairs, upgrades, and generator changes for pacemakers with an ICD or CRT | V53.32, 37.85, 37.86, 37.87, 37.89, 37.96, 37.97, 37.98, 00.53, 00.54 | Z45.02, 02WA0MZ, 0JPT0PZ, 0JWT0PZ, 02PA0MZ, 02PA3MZ, 02WA3MZ, 0JPT3PZ, 0JWT3PZ | 33215, 33218, 33220, 33222, 33223, 33224, 33226, 33234, 33235, 33241, 33244, 33262, 33263, 33264 |
| Revisions, repairs, upgrades, and generator changes for pacemakers without an ICD or CRT | V45.01  V53.31 | Z45.010  Z45.018 | 33212, 33213, 33214, 33221, 33222, 33227, 33228, 33229, 33233, 33234, 33235 |
| Sick sinus syndrome | 427.81 | I49.5 |  |
| Surgical aortic valve replacement | 35.21, 35.22 | 02RF07Z, 02RF08Z , 02RF0JZ, 02RF0KZ | 33405, 33406, 33410, 33411, 33412 |
| Tobacco use | 305.1 | F17.2X |  |
| Transcatheter aortic valve replacement | 35.05, 35.06 | 02RF37Z , 02RF38Z, 02RF3JZ, 02RF3KZ | 33361, 33362, 33363, 33364, 33365, 33367, 33368, 33369 |
| Unspecified cardiac rhythm related device |  | 0JH60PZ, 0JH63PZ, 0JH80PZ, 0JH83PZ |  |
| Valvular disease, aortic insufficiency | 396.1, 396.3 | I06.1, I06.2, I35.1, I35.2, |  |
| Valvular disease, aortic stenosis | 396.0, 396.2 | I06.0 |  |
| Valvular disease, other | 396X | I05X, I06X, I07X, I08X, I09.0, I09.1, I09.8, I34X, I35X, I36X, I37X |  |

*An “X” in the ICD-9 or ICD-10 code represents any possible integer (0-9) that corresponds to more specific descriptions within the listed diagnostic code term.

CRT = Cardiac resynchronization therapy; CRT-D = Cardiac resynchronization therapy defibrillator; ICD = implantable cardioverter-defibrillator; ICD-9 = International Classification of Diseases, Ninth Revision; ICD-10 = International Classification of Diseases, Tenth Revision
