# Supplementary material for: Modifiable predictors of atrioventricular block warranting a pacemaker: A cohort study
Source: PLoS One. 2026 Sep 9;21(9):e0356491. doi: 10.1371/journal.pone.0356491 (PMC13557407; doi:10.1371/journal.pone.0356491)
Supplement: Supplementary file 2 [file pone.0356491.s002.docx]

**S2 Table.** Stepwise Forward Selection of Covariates Responsible For Change From Higher to Lower Risk of Pacemaker Implantation After Multivariable Adjustment in Patients with Atrial Fibrillation

| **Base model** | **Added covariate** | **Cumulative HR (95% CI)** | **P value** |
| --- | --- | --- | --- |
| Atrial fibrillation |  | 11.64 (11.48 – 11.81) | <.001 |
|  | Sick sinus syndrome | 3.83 (3.75 – 3.91) | <.001 |
|  | Age | 1.40 (1.38 – 1.43) | <.001 |
|  | Hypertension | 1.06 (1.06 – 1.06) | <.001 |
|  | Male | 1.20 (1.18 – 1.22) | <.001 |
|  | Valvular disease, other | 1.09 (1.07 – 1.11) | <.001 |
|  | Congestive heart failure | 0.85 (0.83 – 0.86) | <.001 |
|  | Obesity | 0.84 (0.82 – 0.85) | <.001 |
|  | Coronary atherosclerosis | 0.82 (0.80 – 0.83) | <.001 |
|  | Chronic kidney disease | 0.81 (0.80 – 0.83) | <.001 |
|  | Diabetes | 0.82 (0.81 – 0.84) | <.001 |
|  | Atrial flutter | 0.76 (0.74 – 0.77) | <.001 |
|  | Race and ethnicity | 0.75 (0.74 – 0.77) | <.001 |
|  | PM_2.5_ | 0.75 (0.74 – 0.77) | <.001 |
|  | Cannabis use | 0.75 (0.74 – 0.77) | <.001 |
|  | Income | 0.75 (0.74 – 0.77) | <.001 |
|  | Valvular disease, aortic insufficiency | 0.75 (0.74 – 0.76) | <.001 |
|  | Tobacco use | 0.75 (0.74 – 0.76) | <.001 |
|  | Valvular disease, aortic stenosis | 0.75 (0.73 – 0.76) | <.001 |

CI = confidence interval; HR = hazard ratio; PM_2.5_ = particulate matter 2.5.
