# Supplementary material for: Modifiable predictors of atrioventricular block warranting a pacemaker: A cohort study
Source: PLoS One. 2026 Sep 9;21(9):e0356491. doi: 10.1371/journal.pone.0356491 (PMC13557407; doi:10.1371/journal.pone.0356491)
Supplement: Supplementary file 3 [file pone.0356491.s003.docx]

**S3 Table.** Sensitivity Analyses Comparing the Hazard Ratios of Pacemaker Implantation When Including All Patients vs Excluding Patients With Sick Sinus Syndrome vs Excluding Patients With Atrial Fibrillation

|  | **All patients** | | **No SSS** | | **No AF** | |
| --- | --- | --- | --- | --- | --- | --- |
| **Characteristics** | HR | P value | HR | P value | HR | P value |
| Age, y | 1.06 | <.001 | 1.07 | <.001 | 1.07 | <.001 |
| Male | 1.64 | <.001 | 1.70 | <.001 | 1.65 | <.001 |
| Race and ethnicity |  |  |  |  |  |  |
| White | ref | --- | ref | --- | ref | --- |
| Black | 0.97 | 0.03 | 0.94 | 0.001 | 0.87 | <.001 |
| Hispanic | 1.01 | 0.14 | 1.03 | 0.007 | 1.01 | 0.50 |
| Asian and Pacific Islander | 0.83 | <.001 | 0.83 | <.001 | 0.77 | <.001 |
| American Indian and Alaskan Native | 1.01 | 0.77 | 1.03 | 0.60 | 1.07 | 0.22 |
| Other | 1.30 | <.001 | 1.32 | <.001 | 1.39 | <.001 |
| Income quartile |  |  |  |  |  |  |
| 1 (lowest) | 0.93 | <.001 | 0.90 | <.001 | 0.90 | <.001 |
| 2 | 0.95 | <.001 | 0.93 | <.001 | 0.93 | <.001 |
| 3 | 0.97 | 0.004 | 0.97 | 0.003 | 0.97 | 0.006 |
| 4 (highest) | ref | --- | ref | --- | ref | --- |
| Comorbidities |  |  |  |  |  |  |
| Hypertension | 3.26 | <.001 | 3.21 | <.001 | 3.32 | 0.21 |
| Diabetes | 1.33 | <.001 | 1.31 | <.001 | 1.34 | <.001 |
| Atrial fibrillation | 0.75 | <.001 | 0.88 | <.001 |  | <.001 |
| Atrial flutter | 1.54 | <.001 | 1.87 | <.001 | 2.31 | 0.004 |
| Sick sinus syndrome | 10.72 | <.001 |  |  | 16.58 | <.001 |
| Congestive heart failure | 1.75 | <.001 | 2.04 | <.001 | 2.12 | <.001 |
| Coronary atherosclerosis | 1.48 | <.001 | 1.70 | <.001 | 1.56 | <.001 |
| Myocardial infarction | 1.04 | <.001 | 1.03 | 0.01 | 0.99 | <.001 |
| Valvular disease, aortic insufficiency | 1.17 | <.001 | 1.41 | <.001 | 1.36 | <.001 |
| Valvular disease, aortic stenosis | 1.15 | <.001 | 1.39 | <.001 | 1.53 | <.001 |
| Valvular disease, other | 2.19 | <.001 | 2.47 | <.001 | 2.60 | <.001 |
| Chronic kidney disease | 1.38 | <.001 | 1.47 | <.001 | 1.42 | <.001 |
| Lifestyle factors |  |  |  |  |  |  |
| Obesity | 1.48 | <.001 | 1.47 | <.001 | 1.55 | <.001 |
| Alcohol use | 1.08 | <.001 | 1.03 | 0.13 | 1.07 | <.001 |
| Tobacco use | 1.06 | <.001 | 1.03 | 0.04 | 1.07 | <.001 |
| Cannabis use | 1.44 | <.001 | 1.44 | <.001 | 1.47 | <.001 |
| PM_2.5_, μg/m3 | 0.99 | <.001 | 0.99 | <.001 | 0.99 | <.001 |

The models were adjusted and should be interpreted as described in **Fig 2**.

AF = atrial fibrillation; HR = hazard ratio; PM_2.5_ = particulate matter 2.5; SSS = sick sinus syndrome
